# The Sound of Danger: Threat Sensitivity to Predator Vocalizations, Alarm Calls, and Novelty in Gulls

**DOI:** 10.1371/journal.pone.0082384

**Published:** 2013-12-06

**Authors:** Sarah A. MacLean, David N. Bonter

**Affiliations:** 1 Department of Natural Resources, Cornell University, Ithaca, New York, United States of America; 2 Cornell Lab of Ornithology, Ithaca, New York, United States of America; University of Salamanca- Institute for Neuroscience of Castille and Leon and Medical School, Spain

## Abstract

The threat sensitivity hypothesis predicts that organisms will evaluate the relative danger of and respond differentially to varying degrees of predation threat. Doing so allows potential prey to balance the costs and benefits of anti-predator behaviors. Threat sensitivity has undergone limited testing in the auditory modality, and the relative threat level of auditory cues from different sources is difficult to infer across populations when variables such as background risk and experience are not properly controlled. We experimentally exposed a single population of two sympatric gull species to auditory stimuli representing a range of potential threats in order to compare the relative threat of heterospecific alarm calls, conspecific alarms calls, predator vocalizations, and novel auditory cues. Gulls were able to discriminate among a diverse set of threat indicators and respond in a graded manner commensurate with the level of threat. Vocalizations of two potential predators, the human voice and bald eagle call, differed in their threat level compared to each other and to alarm calls. Conspecific alarm calls were more threatening than heterospecfic alarm calls to the larger great black-backed gull, but the smaller herring gull weighed both equally. A novel cue elicited a response intermediate between known threats and a known non-threat in herring gulls, but not great black-backed gulls. Our results show that the relative threat level of auditory cues from different sources is highly species-dependent, and that caution should be exercised when comparing graded and threshold threat sensitive responses.

## Introduction

Prey organisms are faced with the challenge of responding to varying degrees of predation threat in a way that maximizes the benefits of deterring potential predators while also minimizing the costs of time, energy, and risk accrued by employing anti-predator behaviors [1]. The ability of organisms to assess the degree of predation risk and respond with appropriate intensity is referred to as the threat sensitivity hypothesis [2], [3]. Predator recognition can occur through innate and learned mechanisms [4], [5]. Organisms can learn to recognize predator cues through direct experience or observation of conspecifics [6]–[8], and may modify perceived predation risk based on experience over time [9].

The threat sensitivity hypothesis has been well supported for multiple taxa, primarily in the visual and chemical modalities [10]–[12]. Apart from a rich literature on the use of context-specific and graded alarm calls [13]–[17], threat sensitivity has received relatively little attention in the auditory modality. A key question is how the source of an auditory cue influences the predation risk perceived by a receiving organism. In this paper we will consider four general sources of auditory cues: known predator vocalizations, conspecific alarm calls, heterospecific alarm calls, and novel cues. Few previous studies involving novel auditory stimuli exist, the most notable being recent work on responses to unfamiliar heterospecific alarm calls [23], [25]. We were unable to find any studies that evaluate threat perception of a nonthreatening but novel auditory cue. A comparable study from the literature on chemical threat sensitivity found that greater sirens (*Siren lacertian*) respond cautiously to novel cues, but that cues from known predators elicit more intense responses [12].

Among the auditory cues we hypothesize that a more direct source (vocalizations of known predators) will be perceived as a greater threat. Therefore, we predict that predator vocalizations will be more threatening than conspecific alarm calls, which in turn will be more threatening than heterospecific alarm calls. This relationship, however, does not hold across systems. Barrera et al. found that zenaida doves (*Zenaida aurita*) increased vigilance and suppressed foraging more in response to predator playbacks than to conspecific wing whistles, a type of alarm signal [18]. Other studies have found this relationship to be completely reversed. Vigilance increased more after playbacks of conspecific alarm calls than after playbacks of predator vocalizations when presented to both American coots (*Fulica americana*) [19] and yellow-bellied marmots (*Marmota flaviventris*) [20]. Responses to heterospecific alarm calls are less vigorous than responses to conspecific alarm calls for some species pairs [21]–[23], but other species respond equivalently to both heterospecific and conspecific alarm calls [23], [24].

Threat sensitivity is determined by more than just the source of a threat, and failure to control for these additional factors could explain why we see apparently contradictory results across studies. The level of background risk impacts the intensity of response to both known and novel threat stimuli [5], [26]. Group size has also been shown to affect the way organisms respond to threats [27]. For alarm calls, responses are mediated by signal reliability, and a signal consistently given in the absence of a threat is less likely to be heeded [28], [29] Reliability may be evaluated on an individual basis [28], or in the case of conspecific versus heterospecific calls, between species. Heterospecific alarm calls vary in reliability across species, and this reliability depends on how vulnerable the sender species is to predators as well as whether this vulnerability is shared equally by the receiving species [24]. For example, if we consider two similar species that differ in size, the smaller species is likely vulnerable to the same threats as the larger species, but the reverse may not be true.

Finally, threat sensitivity can manifest as graded antipredator behavior that is directly proportional to the perceived threat, or an organism may display a threshold or “hypersensitive’ response, in which all stimuli above a certain threat level elicit a response of similar intensity [3], [8], [27]. The hypothesis that predator vocalizations will always be perceived as more threatening than alarm calls suggests a type of hypersensitive response. We believe that the conflicting results in the literature are better explained by graded responses, in which organisms perceive different threat levels associated with different species of predators and with alarm calls from different sources. Assuming that certain predators are more threatening than others, then some predator vocalizations will be perceived as more threatening than alarm calls, but not others [30].

In order to isolate the effect of cue source on auditory threat sensitivity, a diverse gradient of potential threats must be presented to individuals within a single and relatively homogenous population. We conducted an auditory playback experiment on isolated, nesting individuals of two sympatric species of gull in a small archipelago in the Gulf of Maine. Our two focal species share similar life histories, but vary in size and therefore vulnerability to predation. We predicted that (1) gulls would respond to varying degrees of predation threat in a graded manner, (2) predator vocalizations from different species would elicit reactions of different intensity, (3) a novel sound would elicit a reaction intermediate between know threats and a known non-threat, and (4) conspecific alarm calls would elicit more intense responses than heterospecific alarm calls in the larger focal species, but both call types would be equally threatening to the smaller species.

## Methods

### Ethics Statement

This research was carried out in strict accordance with the Guidelines for the Use of Wild Birds in Research of the Ornithological Council and approved by the Cornell University Institutional Animal Care and Use Committee (protocol #2011-0036). Research was conducted on property managed by Cornell University for research and educational purposes. This work did not involve any threatened, endangered or protected species.

### Study Site and Species

We studied great black-backed gulls and herring gulls nesting on the Isles of Shoals Archipelago (42.98°, –70.61°), approximately 11 km offshore of Portsmouth, New Hampshire, USA. Playback experiments were conducted on Appledore and Smuttynose Islands. Nesting density is highest along the rocky shorelines of the islands and lower in the interior, where birds nest in loose subcolonies around human settlements and are relatively isolated from congenors [31]. In order to control for greater acclimation to humans, limit the influence of neighboring birds on individual reactions, and avoid repeatedly exposing birds to test stimuli, we only tested isolated gulls in the loose subcolony setting.

The only natural nest predators in our study population are large raptors and other gulls. Although great black-backed gull predation on herring gull nests is common [32], both conspecific and heterospecific nest predation has been observed for both study species. Despite some acclimation to human presence, gulls in the Shoals Archipelago have not become habituated to humans, and in fact ferociously attack any human in the vicinity of their nest.

Gulls exhibit a wide range of highly stereotyped anti-predator behaviors requiring varying amounts of energy and physical risks [33]. As such, this is an ideal study system for testing predictions of the threat sensitivity hypothesis. Adult herring gulls average 800–1250 g in mass with up to a 66 cm wingspan [34], while great black-backed gulls are significantly larger, with an average mass of 1300–2000 g and up to a 79 cm wingspan [35]. Great black-backed gulls nesting on Appledore Island are more aggressive in nest defense than their smaller counterparts [32].

### Stimulus Selection

Playbacks included six auditory stimuli. Predator vocalizations included the call of a bald eagle (*Leucocephalus haliaeetus*) and a recording of a human voice. Colonial birds are a major food source for bald eagles in Maine [36]. However, in our study system, gulls are much more regularly exposed to human “predators.” People historically posed a large threat to nesting gulls by collecting both eggs and adult birds for food [37], and to this day some people illegally shoot at gulls or intentionally destroy nests to discourage nesting on their property. Alarm calls included recordings of the ‘*yeow*’ call of both the herring gull and great black-backed gull. The ‘*yeow’* call is a stereotyped alarm call shared by a large number of gull species. Reaction to a novel stimulus was tested using the *‘weep’* call of a western scrub-jay (*Aphelocoma californica*), a passerine native to western North America, which gulls nesting in Maine are unlikely to have encountered previously. This call is used as a contact call or for territorial displays, and was chosen because it bears minimal structural similarity to any avian call familiar to Maine gulls. The song of a song sparrow (*Melospiza melodia*), a small passerine that is abundant in the Shoals Archipelago, was a familiar, non-threatening sound used as a control.

All recordings were obtained from the Macaulay Library at the Cornell Lab of Ornithology (www.macaulaylibrary.org), with the exception of one herring gull recording that was recorded on Appledore Island in summer 2010. The human voice tracks included 30 sec readings of a book passage by three different female individuals and were recorded in the Macaulay Library sound studio. In order to control for signal strength, all recordings were equalized to playback at similar peak volumes.

In order to obtain a baseline for the upper limit to intensity of reactions, we also tested reactions to a visual stimulus in the form of a human approaching and standing 1 m from the nest. Gulls are largely visually-biased organisms, meaning that in the open areas where they nest, they are likely to see a predator before detecting it via alternate sensory modalities [38]. Therefore, seeing a large predator such as a human approach their nest represents one of the greatest threats these birds could potentially encounter.

### Playback Experiment

We presented playbacks to incubating herring gulls and great black-backed gulls during May and June of 2011. Recordings were played using an iPod connected to portable speakers (iHome model iHM11), which were placed approximately 1 m from the nest. Only incubating birds were tested, and each nest received all stimuli only once. During the auditory playbacks, the observer remained hidden behind natural barriers.

To control for possible bias introduced by individual recordings, three versions of each auditory stimulus were used, representing independent recordings of different individuals. To control for stimulus order, six playlists were constructed, each containing all six stimuli in a randomized order. Playlists also helped control for observer bias during playbacks, since the observer did not know the order in which tracks were presented and observed from a sufficient distance that playbacks were not readily audible (exact distance varied according to the availability of natural barriers). Each track version for each stimulus was used in two separate playlists. Gulls never leave their nests unattended, so each playlist began with 3 minutes of silence to allow the focal bird to return to a calm state after the observer placed the playback equipment near the nest. Stimuli were then played for 30 sec each, with 2 minutes of silence between each stimulus to allow birds to return to a calm state. This was always sufficient time for the birds to visibly return to a calm state (a 0 on the rating scale in Table 1) while sitting on their nests.

**Table 1:** Categorical values used to rate the overall reaction of gulls to auditory and visual stimuli.

Response Value*	Description of Response
0	No response
1	Initial increase in vigilance followed by relaxation during 30 sec period
2	Increased vigilance for entire 30 sec period: neck slightly raised, casual scanning for threats, does not interrupt activities like panting
3	Extremely vigilant for entire 30 sec period: neck fully outstretched, rapid scanning for threats, activities like panting often interrupted, use of ‘*kek-kek’* calls
4	Use of ‘*yeow*’ alarm call
5	Stood up off eggs
6	Movement within 3 m of nest
7	Movement beyond 3 m of nest, including flight

* Higher values represent more energy-intensive responses and may also include behaviors described under lower response values.

The visual stimulus was tested either before or after the auditory playbacks wherein the observer stood 1 m from the nest for 30 sec. When the visual stimulus was tested before the auditory stimuli, the 3 minutes of silence at the beginning of each playlist allowed birds to return to a calm state while the observer left the area and hid from view of the focal bird. When the visual stimulus was tested after the auditory stimuli, the observer waited 2 minutes after the last auditory stimulus ended before approaching the nest.

For each stimulus tested (auditory and visual), the maximum response of the bird was recorded using a categorical scale ranging from 0–7 (Table 1). Each number corresponded to a discrete set of behaviors, based on the descriptions of Tinbergen [33] and personal observations. After each stimulus ended, the time in seconds before the bird returned to a calm state was estimated. This latency was recorded categorically as 0–3 sec, 4–15 sec, 16–30 sec, 31–45 sec, 46–60 sec, or >60 sec. Categorical variables were used due to the difficulty of determining the exact moment that a bird returned to a calm state.

### Statistical Analysis

Differences in the reactions of gulls to various stimuli presented by the experiment were quantified using generalized linear models for categorical response variables in SAS version 9.2 (PROC GENMOD, SAS Institute 2008). In addition to stimulus type (*N* = 7, audio playback or physical approach), the order in which each stimulus was presented (order, *N* = 7) and track version number (*N* = 3 for each auditory stimulus, nested within stimulus) were included in the model. Because each individual bird was presented with all 7 stimuli, individual was used as a random (repeated) variable in the models. We computed log odd ratios comparing all pairs of stimuli in order to test for differences in the intensity of reaction to the various treatments. Two response variables were analyzed: maximum response (reaction), and the time that the bird remained vigilant following the stimulus (latency). Because latency was skewed toward short periods of vigilance, models did not converge when the variables order and track number were included in these models. Therefore, stimulus was the only explanatory variable included in the latency models. Separate models were run for each species.

In order to test for overall differences in intensity of reaction between the two focal species, we built a separate model that include stimulus type, order, version (nested within stimulus), species, and a species by stimulus interaction.

## Results

### Herring Gull

**Figure 1:**
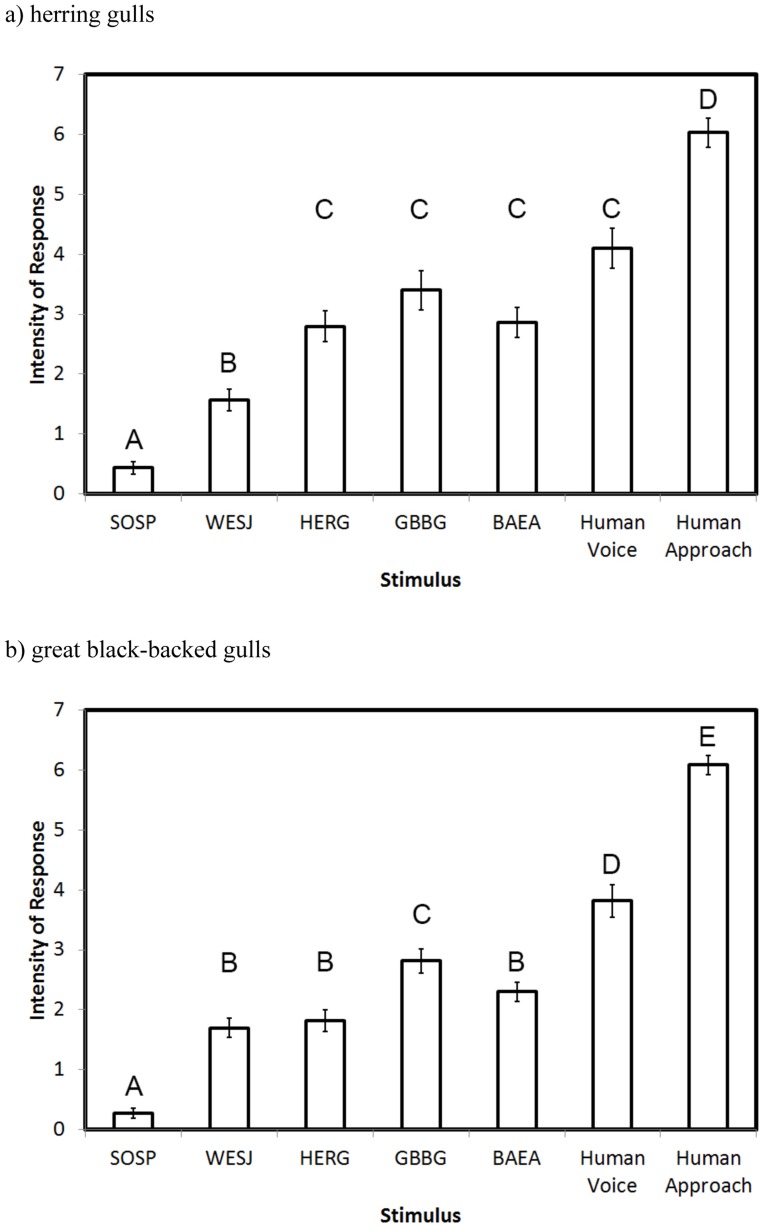
Intensity of response to auditory threats. Intensity of response by (a) herring gulls and (b) great black-backed gulls to various auditory stimuli and a physical approach by a human. Raw means and standard errors are reported, with different letters indicating statistically significant differences among stimuli, based on log odds ratios. SOSP  =  song sparrow, WESJ  =  western scrub-jay, HERG  =  herring gull, GBBG  =  great black-backed gull, BAEA  =  bald eagle.

Thirty incubating herring gulls were exposed to the series of auditory stimuli and a physical approach. Individuals showed significant differences in the intensity of response to the various stimuli (*F*_6,210_ = 28.84, *P*<0.001; Fig. 1a). Birds showed minimal reaction to playbacks of song sparrow vocalizations, the control sounds used in the experiment, suggesting that playback volume or speaker quality were not artificially influencing gull reactions. The novel call of a western scrub-jay elicited a response stronger than the song sparrow but less than any other auditory stimuli (*P*<0.001). Reactions to herring gulls alarm calls, great black-backed gull alarm calls, bald eagle calls, and the human voice were elevated and not significantly different from one another. The visual approach by a human elicited a stronger reaction than any of the auditory stimuli (*P*<0.05). Neither order in the playlist (*F*_6,210_ = 10.34, *P* = 0.111) nor version of each auditory stimuli (*F*_2,210_ = 12.64, *P* = 0.396) explained variation in the intensity of response.

**Figure 2:**
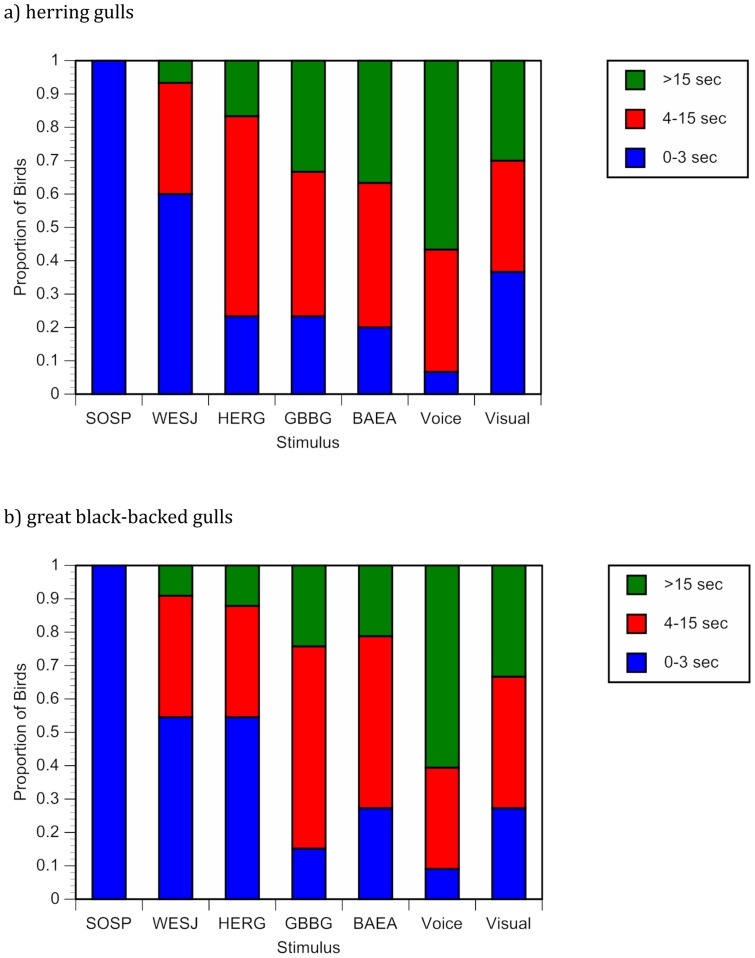
Duration of response to auditory threats. Duration of response by (a) herring gulls and (b) great black-backed gulls to various auditory stimuli and a physical approach by a human. Because the majority of responses fell in lower time intervals, latencies above 15 sec have been binned to aid visualization. SOSP  =  song sparrow, WESJ  =  western scrub-jay, HERG  =  herring gull, GBBG  =  great black-backed gull, BAEA  =  bald eagle.

Differences were also detected in the duration of the reaction to the stimuli (*F*_6,210_ = 26.58, *P*<0.001; Fig. 2a). Following the playback of the human voice, birds remained agitated for significantly longer than any of the other stimuli tested, including the physical approach by a human. The physical approach, alarm calls of both species, and vocalization of the bald eagle all elicited similar periods of vigilance. The call of the western scrub-jay elicited a period of vigilance that was significantly less than any of the known threat stimuli, but greater than the song sparrow control.

### Great Black-backed Gull

Thirty-three incubating great black-backed gulls were exposed to the same series of stimuli presented to the herring gulls. As with their congeners, great black-backed gulls showed a wide range of responses to the various stimuli (*F*_6,231_ = 29.19, *P*<0.001; Fig. 1b) while order of the stimulus (*F*_6,210_ = 12.18, *P* = 0.058) and stimulus version (*F*_2,210_ = 15.84, *P* = 0.199) did not influence reactions. The human approach instigated the most vigorous responses (*P*<0.001), and playbacks of the human voice were perceived as the most threatening of the auditory stimuli (*P*<0.05). Whereas herring gulls reacted strongly to the alarm calls of the larger great black-backed gull, the inverse relationship was weaker. Great black-backed gulls reacted more strongly to great black-backed gull alarm calls than to herring gull alarm calls (*P*<0.001). Reactions to conspecific alarm calls were also greater than reactions to bald eagle calls (*P* = 0.003) and the novel calls of the western scrub-jay (*P* = 0.001). Reactions to herring gulls alarm calls, bald eagle calls, and the western scrub-jay calls were not significantly different from one another.

Duration of the reaction to various stimuli varied (*F*_6,231_ = 29.75, *P*<0.001); as with herring gulls, great black-backed gulls remained vigilant longer after hearing the human voice than following exposure to any of the other stimuli (Fig. 2b). The physical approach, great-black backed gull alarm call, and bald eagle vocalization elicited similar periods of vigilance. Significantly shorter periods of vigilance were elicited by playbacks of the western scrub-jay call and herring gull alarm call.

For the model including both species, reaction intensity was not significantly affected by species (*F*_1,441_ = 0.45, *P*  = 0.501), nor did we detect a species by stimulus interaction (*F*_6,441_ = 5.20, *P* = 0.519).

## Discussion

Gulls responded to auditory playbacks of a wide range of auditory stimuli in a graded manner, commensurate with the level of threat. Our results also show that more direct sources of information about predation risk (predator vocalizations, for example) are not always perceived as a greater threat. Instead, the relative threat level of predator vocalizations, alarm calls, and even novel stimuli varies according to the species of both sender and receiver.

### Response to the Visual Stimulus

Surprisingly, while the approach of a human elicited a more intense overall response than did the playback of a human voice, birds took significantly longer to return to a calm state after exposure to the latter. We believe this was due to the greater uncertainty associated with the auditory stimulus. Because the origin of the human voice could not be determined, the cessation of the playback did not necessarily indicate that a threat was no longer nearby. In contrast, when a human was visible near the nest, birds could identify the exact location and behavior of the threat. Being able to see a threatening organism leave the area seems to serve as a more reliable indication that the threat is indeed gone, especially given the relatively open habitat where gulls typically nest.

### Response to Predator Vocalizations

In great black-backed gulls, the human voice recording elicited the strongest response of any auditory stimulus. The call of the bald eagle, also a predator vocalization, elicited a response roughly similar to a herring gull alarm call, but less than a great black-backed gull alarm call. This result demonstrates that predator vocalizations vary in their perceived threat level, which can in turn affect whether a predator vocalization is perceived as more threatening than an alarm call. A similar result was found in a study of black-casqued hornbills (*Ceratogymna atrata*), where birds responded more strongly to predator cues than alarm calls when the predator being indicated was an eagle, but not when the predator was a leopard [30]. We were still surprised, however, to see a great black-backed gull call elicit a stronger reaction than an eagle call, especially given anecdotal evidence that the visual presence of a large raptor or even an airborne object such as a toy kite causes significant disturbance within the colony. The most likely explanation is that eagles do not often vocalize while hunting [20], and gulls are more attuned to the sight of a bald eagle than to its vocalizations. When predators vocalize frequently, as is the case with humans, then their vocalizations serve as more reliable indicators of presence and will be more likely to elicit a response from prey [18].

Herring gulls responded with similar intensity to both predator vocalizations and alarm calls. Herring gulls seem to have perceived all these stimuli as so threatening that no discernible difference was evident among them. This result could be interpreted as a hypersensitive rather than graded response, if not for our inclusion of the human approach, which elicited a significantly more intense reaction than these four auditory threat stimuli. It is possible, therefore, that an organism display a seemingly hypersensitive response to a subset of stimuli, but the response to a wider gradient of stimuli is still a graded one. The identification of a threat sensitivity threshold might, then, be an artifact of the range of threat stimuli tested in some situations. This should be an important consideration in future studies of graded versus hypersensitive threat sensitivity.

### Response to Conspecific and Heterospecific Alarms

The relative threat level associated with conspecific as opposed to heterospecific alarm calls varied by species. Great black-backed gulls reacted more strongly to conspecific alarms than to alarms of the herring gull, whereas herring gulls gave equal credence to both. Although both gulls recognize and respond to each other’s alarm calls, the information encoded in these heterospecific alarms is asymmetric. Reliability has been cited as a key component of such asymmetry, meaning that the alarm calls of some species accurately indicate the presence of a predator with greater frequency than do others [39]. New Holland honeyeaters (*Phylidonyris novaehollandiae*) presented with the alarm calls of two sympatric species responded more intensely to the alarm call of a scrubwren (*Sericornus frontalis*), which indicated danger with greater accuracy and therefore reliability [24]. In that example, asymmetry existed from the perspective of one species responding to alarm calls of multiple heterospecifics. Our study demonstrates that similar asymmetry in reliability can exist even within a species pair. A herring gull will be vulnerable to whatever threatens a great black-backed gull, but the larger and more aggressive great black-backed gull will be vulnerable to only a subset of whatever threatens a herring gull. Because great black-backed gulls occasionally prey on herring gull nests, the alarm call of a great black-backed gull could also be interpreted as a predator vocalization by a herring gull. In addition to the interspecific variation in alarm call reliability documented by our study, variation in signal reliability can also exist within a species, when organisms respond with greater intensity to calls of certain individuals or age classes [40].

### Response to a Novel Stimulus

Playbacks of the western scrub-jay call elicited responses that were intermediate between known threats and the non-threatening control when presented to herring gulls. This result is similar to that found in studies of novel chemical cues [12], and satisfies our initial predictions. However, great black-backed gulls responded with similar intensity to the western scrub-jay call, the herring gull alarm call, and the bald eagle call, suggesting that novel auditory stimuli can be perceived as having a threat level comparable to that of known threat stimuli. This could be due to either low sensitivity (compared to herring gulls) of great black-backed gulls to herring gull and bald eagle calls, or unexpectedly high sensitivity by these birds to the novel stimulus.

Auditory neophobia has been documented in only a handful of studies, notably for domestic animals [41], [42], and has yet to be sufficiently explored. Responses to novel auditory cues have mainly been investigated in the context of learning and aversive conditioning [43]–[45]. We were unable to find any previous studies of neophobia to novel auditory cues in birds. Furthermore, we were able to find only one study of primates that compared responses to novel auditory stimuli with those elicited by familiar stimuli in a natural setting [46]. Investigation of auditory neophobia in a meaningful ecological context – how organisms interpret and respond to novel sounds in their environment – is severely lacking. Our study demonstrates that two very similar species can have different relative reactions to the same novel auditory stimulus. Further investigation of auditory neophobia in other taxa and with different types of novel stimuli will yield more valuable insights to animal communication and threat perception.

Although we chose a visual stimulus to represent our baseline for maximum potential threat from the perspective of a nesting gull, the relative importance of visual information is likely reduced in situations where visual predator recognition is limited, such as for cavity-nesting species [47]. Other sources of sensory information, such as auditory or olfactory cues, might play a greater role in such situations. Although there is a rich literature addressing chemical threat sensitivity in fish and amphibians, little research attention has focused on this topic in birds. Increasing evidence, however, suggests that some bird species are capable of detecting predators by smell [47]–[49]. The family of tubenosed seabirds is particularly well known for using their well-developed olfactory abilities in foraging [50] and identification [51], and odor discrimination abilities have been documented in at least one Larid, the black-legged kittiwake (*Rissa tridactyla*) [52]. Quantifying the relative threat level of stimuli from different sensory modalities, and how these relationships are influenced by the life history of an organism, would be an interesting avenue for future research.
